# Associated factors and a risk prediction model for comorbid circadian rhythm sleep-wake disorders in patients with chronic fatigue syndrome: implications for early screening and sleep health management

**DOI:** 10.3389/fpsyt.2026.1826328

**Published:** 2026-07-20

**Authors:** Xiao Shao, Fan Yang, Le-le Qin, Jia-ning Shi, Min Chen, Wen-jin Ge, Tian-jun Jiang, Jing-wen Yue, Wen Feng, Jing-han Wang, Zhen-xian Zhang

**Affiliations:** 1 Yueyang Hospital of Integrated Traditional Chinese and Western Medicine, Shanghai University of Traditional Chinese Medicine, Shanghai, China; 2 Tongren Hospital, Shanghai Jiao Tong University School of Medicine, Shanghai, China; 3 Shanghai Seventh People’s Hospital of Shanghai University of Traditional Chinese Medicine, Shanghai, China

**Keywords:** chronic fatigue syndrome, circadian rhythm sleep-wake disorders, nomogram, shift work, sleep health

## Abstract

**Objective:**

To characterize comorbid circadian rhythm sleep-wake disorders (CRSWDs) in patients with chronic fatigue syndrome (CFS), identify factors independently associated with CRSWDs, and develop a risk prediction model for early screening and sleep health management.

**Methods:**

This retrospective study included 610 patients with CFS, who were randomly divided into a training cohort (*n* = 427) and a validation cohort (*n* = 183) in a 7:3 ratio. Demographic characteristics, lifestyle factors, and sleep-related variables were analyzed using univariable and multivariable logistic regression. A nomogram was developed on the basis of the final model. Its discriminative ability, calibration, and public health usefulness were evaluated using receiver operating characteristic (ROC) analysis, the Hosmer-Lemeshow goodness-of-fit test, and decision curve analysis (DCA), respectively.

**Results:**

Among the 427 patients with CFS in the training cohort, 208 (48.7%) had comorbid CRSWDs. Univariable analysis showed that gender, BMI, fatigue severity (Fatigue Scale-14, FS-14), slow-wave sleep (SWS, N3%), duration of screen use before bedtime and shift-work schedule type were significantly associated with CRSWDs (*P ≤* 0.05). Multivariable logistic regression analysis identified 12-hour rotating shifts (OR = 6.981, 95% CI: 2.603-18.720), 24-hour shifts (OR = 5.316, 95% CI: 2.197-12.863), 8-hour rotating shifts (OR = 3.982, 95% CI: 1.698-9.339), FS-14 (OR = 1.984, 95% CI: 1.442-2.728) and female sex (OR = 1.892, 95% CI: 1.178-3.037) as independently associated with an increased incidence of CRSWDs. By contrast, an increase in SWS (N3%) was independently associated with decreased odds of CRSWDs (OR = 0.982, 95% CI: 0.971-0.992). The AUCs in the training and validation cohorts demonstrated good model performance (AUC: 0.808, 0.767, respectively), and the Hosmer-Lemeshow test revealed a satisfactory fit.

**Conclusion:**

Female sex, greater fatigue severity, reduced N3 sleep, and rotating shift schedules were independently associated with comorbid CRSWDs in patients with CFS. The model may be useful for early screening and risk stratification and may help inform sleep hygiene education and schedule-related health management in individuals at elevated risk.

## 1 Introduction

CFS is a complex disorder primarily characterized by persistent, debilitating fatigue lasting for more than six months. It is frequently accompanied by multisystem symptoms, including sleep disturbances, cognitive impairment, and autonomic dysfunction, all of which markedly impair patients’ quality of life and social functioning (1). Clinical studies suggest that the onset and progression of CFS are closely associated with CRSWDs (2, 3). Individuals with CFS commonly report difficulty initiating sleep, impaired sleep maintenance, and non-restorative sleep, reflecting underlying circadian dysregulation (4). In CFS, the severity of insomnia has been shown to correlate with the attenuation of circadian rhythm amplitude (5).

Studies have demonstrated that patients with CFS exhibit abnormalities in endogenous circadian markers, such as delayed core body temperature peaks and disrupted phases of melatonin secretion (6). Among the exogenous factors influencing circadian rhythms in patients with CFS, contemporary lifestyles appear particularly important. Shift work is a major contributor (7, 8), as irregular work schedules repeatedly disrupt the synchronizing effects of environmental zeitgebers, thereby altering clock gene expression and desynchronizing the sleep-wake cycle. Such circadian misalignment has been linked to increased risks of multiple chronic conditions (9, 10). In addition, insufficient exposure to natural daylight during the daytime and excessive exposure to artificial light at night may further aggravate circadian disruption (11). Daytime light deprivation attenuates the primary entrainment signals for the circadian system, leading to reduced rhythm amplitude and unstable phase alignment; conversely, blue light emitted from electronic screens markedly suppresses melatonin secretion, delays endogenous circadian phase, and interferes with sleep initiation and maintenance. Prolonged exposure can ultimately lead to persistent circadian rhythm disturbances (12, 13).

However, evidence remains limited regarding the specific roles of shift-work patterns, daytime light exposure, and pre-sleep electronic screen use in the occurrence of CRSWDs among patients with CFS. Clarifying the contribution of these potentially modifiable factors may improve early identification of high-risk individuals and provide a basis for sleep hygiene education and targeted health management. Accordingly, this study aimed to characterize comorbid CRSWDs in patients with CFS, examine the associations of shift-work characteristics, light exposure, and pre-sleep screen use with CRSWDs, and develop a risk prediction model to support early screening, risk stratification and sleep health promotion in this population.

## 2 Materials and methods

### 2.1 Sample size calculation

The minimum sample size required for binary logistic regression was estimated using the formula *N* = 10×*k/p*, following the recommendation of Peduzzi et al. (14). Here, *N* denotes the minimum sample size, *k* the number of predictor parameters, and p the proportion of the outcome of interest. For the model in this study (*k* = 17), based on retrospective data from our department, the estimated outcome incidence was 48.7% (*p* = 0.487). The calculated minimum sample size was 411 participants, confirming that the 427 participants enrolled in this study were sufficient for the planned regression analysis. The participants were randomly divided into a training cohort and a validation cohort using a 7:3 ratio. This split is a widely accepted heuristic in predictive modeling, ensuring sufficient data to train the model effectively while preserving a robust, independent sample for internal validation.

### 2.2 Participants and general data

**Figure 1:**
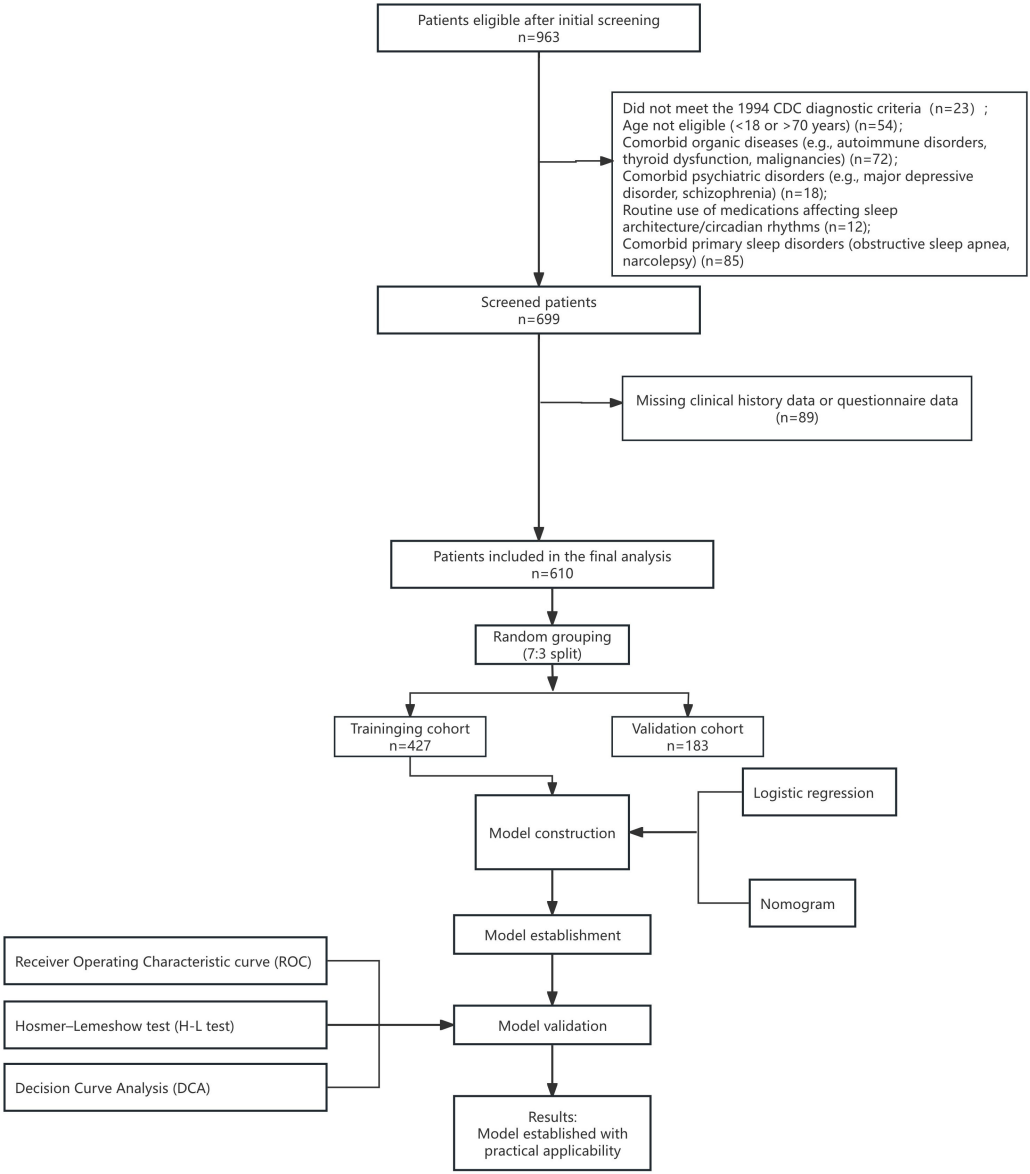
Study flowchart. Study flowchart showing patient screening, inclusion and exclusion, random allocation to the training and validation cohorts, and subsequent model development and validation.

We included 610 patients with CFS who attended Yueyang Hospital of Integrated Traditional Chinese and Western Medicine, Shanghai University of Traditional Chinese Medicine, between January 2023 and June 2025. This study was reviewed and approved by the Ethics Committee of Yueyang Hospital of Integrated Traditional Chinese and Western Medicine, Shanghai University of Traditional Chinese Medicine (approval number: 2025-167). All study procedures were carried out in accordance with the ethical principles of the Declaration of Helsinki and its subsequent revisions. The study flowchart is shown in **Figure 1**. 

#### 2.2.1 Inclusion criteria

Patients met the 1994 U.S. Centers for Disease Control and Prevention (CDC) diagnostic criteria for CFS (15).

Patients were aged 18–70 years.

Complete clinical data were available.

#### 2.2.2 Exclusion criteria

Missing key data in demographic information, physical examination, sleep monitoring, or questionnaire results.

Presence of other identifiable organic diseases (e.g., autoimmune disorders, thyroid dysfunction, malignancies) or psychiatric disorders (e.g., major depressive disorder, schizophrenia, diagnosed via DSM-5 criteria by a psychiatrist).

Presence of primary sleep disorders (e.g., obstructive sleep apnea, narcolepsy).

Routine use of medications known to heavily influence sleep architecture or circadian rhythms (e.g., strong sedatives, hypnotics, melatonin agonists) prior to assessment.

### 2.3 Data collection

This retrospective study included patients diagnosed with CFS. Drawing on preliminary work conducted by our research group and relevant literature (16–18), as well as sleep-monitoring data, we identified the following covariates for analysis: gender, age, overweight/obesity (BMI ≥ 24 kg/m²), FS-14, total sleep time (TST), sleep latency, sleep efficiency (SE), number of awakenings, SWS (N3%), average daily light exposure duration, duration of pre-sleep electronic screen use, history of shift work, total years of shift work, shift rotation frequency (19, 20) (with rapid shift defined as schedule changes within 1 week, medium shift as changes every 1–2 weeks, and slow shift as changes every 3–4 weeks or longer, based on occupational health guidance and relevant studies), type of shift schedule (20) (Regular day shift, Retirement, 8-hour rotating shifts, 12-hour rotating shifts, 24-hour shifts), smoking and alcohol history, and coffee/tea consumption history. These variables were included as covariates to explore factors associated with comorbid CRSWDs among individuals with CFS.

### 2.4 Diagnostic assessment

#### 2.4.1 Case data and clinical measures

We retrospectively reviewed the electronic medical records of patients with CFS who attended the Department of Traditional Chinese Medicine, Yueyang Hospital of Integrated Traditional Chinese and Western Medicine, Shanghai University of Traditional Chinese Medicine. Demographic information, medical history, physical examination findings, previous diagnoses, medication history over the 3 months prior to assessment, and relevant laboratory or imaging findings were extracted from electronic medical records. Medication history was independently reviewed by two experienced clinicians and categorized into: (1) strong sedatives/hypnotics; (2) antidepressants; (3) melatonin agonists; (4) antihistamines with sedative effects; and (5) other medications known to influence sleep architecture or circadian rhythms. Patients with routine use (defined as ≥3 times per week for ≥2 consecutive weeks) of medications in categories (1–3) were excluded from the study, as specified in the exclusion criteria. Key data collected included scores from the FS-14, Pittsburgh Sleep Quality Index (PSQI), Morningness-Eveningness Questionnaire (MEQ-SA), patient sleep diaries, a shift-work history questionnaire, and questionnaires assessing daily light exposure and pre-sleep electronic screen time. Sleep diary information included habitual bedtime and wake-up time, sleep onset latency, nocturnal awakenings, TST, daytime sleepiness or dysfunction, and irregular sleep-wake schedules. Shift-work information included shift-work history, total years of shift work, shift rotation frequency, and shift schedule type.

For patients with available sleep-monitoring records, overnight polysomnography or portable sleep monitoring was performed using an Embletta MPR PG system (Embla Systems, Kanata, Ontario, Canada). Sleep-related parameters used in the present analysis were extracted from existing clinical sleep-monitoring reports, including TST, SE, sleep latency, rapid eye movement (REM) sleep latency, wake after sleep onset (WASO), number of awakenings, and SWS (N3%). All extracted data were independently reviewed and verified by two experienced clinicians to ensure accuracy.

#### 2.4.2 CFS diagnostic criteria

CFS was diagnosed in accordance with the 1994 CDC definition (Fukuda criteria) (15). The 1994 Fukuda criteria were utilized due to their high specificity and extensive validation in identifying CFS in retrospective epidemiological cohorts, ensuring consistency with historical legacy data. Diagnosis required persistent or recurrent fatigue of new or definite onset that was unexplained, not attributable to ongoing exertion, not substantially relieved by rest, and accompanied by a substantial reduction in prior levels of activity. Patients were also required to have at least four concomitant symptoms persisting for 6 months or longer, with these symptoms occurring after the onset of fatigue:

1. Significant impairment in short-term memory or concentration (causing substantial reduction in occupational, educational, social, or personal activities compared to pre-illness level).
2. Sore throat.
3. Tender cervical or axillary lymph nodes.
4. Muscle pain.
5. Multi-joint pain without joint swelling or redness.
6. Headaches of a new type, pattern, or severity.
7. Unrefreshing sleep.
8. Post-exertional malaise lasting more than 24 hours.

#### 2.4.3 CRSWDs diagnostic criteria

Diagnosis of CRSWDs was independently made by board-certified sleep medicine specialists in strict accordance with the International Classification of Sleep Disorders, Third Edition, Text Revision (ICSD-3-TR) (21), and related expert consensus guidelines (22, 23), and all diagnostic records were cross-verified by a second senior sleep clinician to ensure consistency. Specific CRSWD subtypes, such as shift work disorder (SWD) and delayed sleep-wake phase disorder (DSWPD), were identified when applicable. According to the ICSD-3-TR, CRSWDs are characterized by persistent or recurrent sleep-wake disturbances caused by alterations in the endogenous circadian timing system or misalignment between the endogenous circadian rhythm and the required sleep-wake schedule.

Patients were considered to have a CRSWD if they had a chronic pattern of a significantly delayed or advanced sleep onset time (≥ 3 months), or a sleep-wake schedule misaligned with normal societal times, and met any one of the following criteria (indicative of a circadian rhythm abnormality):

Circadian rhythm disturbance on sleep monitoring: Polysomnography or at least 7 days of sleep diary demonstrates a weakened circadian activity rhythm, characterized by reduced amplitude, a phase delay or advance of ≥ 2 hours relative to normal, or frequent nocturnal awakenings (≥ 2 times per night, as defined in ICSD-3-TR).

Chronic maladaptive sleep schedule: A history of night-shift work (between 00:00–06:00, ≥ 3 nights per week for ≥ 1 year) or a long-term habit of late bedtime (sleep onset later than 24:00, ≥ 3 nights per week for ≥ 1 year), or the need to engage in travel across time zones for work (with resultant insomnia or daytime sleepiness, reduced TST, daytime dysfunction, malaise or other physical symptoms, occurring at least once per year), leading to insomnia symptoms or excessive daytime sleepiness with impairment in daytime function.

Extreme chronotype on questionnaire: Score on the MEQ-SA (24) ≤ 41 (indicative of an extreme “evening type”) or ≥ 59 (extreme “morning type”).

To avoid circularity during our predictive modeling, the diagnosis of CRSWDs utilized as the outcome variable in the logistic regression was established strictly using objective circadian rhythm disturbances on polysomnography/sleep diaries and extreme chronotypes on the MEQ-SA. The criterion of “chronic maladaptive sleep schedule” (shift-work history) was intentionally excluded from the outcome definition in the regression analysis to ensure that shift work as a predictor was not artificially correlated with the diagnostic outcome.

### 2.5 Statistical analysis

Data were analyzed using SPSS 25.0. According to the Kolmogorov-Smirnov (K-S) test, continuous variables were not normally distributed and are therefore presented as the median, *M* (*P_25_*, *P_75_*). Categorical variables are expressed as frequencies and percentages. Participants were grouped according to the presence of circadian rhythm disruption, and differences in demographic characteristics, clinical scale scores, and sleep architecture parameters were compared between groups. The Mann-Whitney *U* test was used for comparisons between two groups, the *H* test was used for comparisons among multiple groups, and the *χ²* test was used for comparisons of categorical variables. Based on the univariable analyses, a binary logistic regression model was constructed; results are reported as odds ratios (ORs) with 95% confidence intervals (CIs). Multicollinearity among independent variables was assessed using the Variance Inflation Factor (VIF). A two-sided significance level of *α* = 0.05 was applied.

A nomogram was generated in RStudio using the rms and caret packages. Discrimination and calibration were assessed in both the training and validation cohorts by ROC analysis and the Hosmer-Lemeshow test, respectively. Predictive accuracy was also estimated. The potential clinical value of the model was then examined using DCA.

## 3 Results

### 3.1 Descriptive characteristics

A total of 610 patients with CFS were included in this retrospective study. In the training cohort (*n* = 427), 208 individuals (48.7%) were diagnosed with CRSWDs. Comparative analyses showed that, relative to the non-CRSWDs group, the CRSWDs group differed significantly (*P* < 0.05) across multiple variables, including gender, overweight and obesity, FS-14, average daily light exposure, pre-sleep screen duration, frequency of awakenings, SWS (N3%), total shift work duration, shift-work schedule types and shift-work rotation frequency. By contrast, no significant intergroup differences (*P* > 0.05) were observed in age, smoking/alcohol habits, coffee/tea consumption, total sleep duration, SE, sleep latency or REM sleep latency. Detailed results are presented in **Table 1**.

**Table 1:** General characteristics of CFS patients (model training cohort) with and without comorbid CRSWDs.

Variables	Total (*n* = 427)	Non-CRSWDs (*n* = 219)	CRSWDs (*n* = 208)	*Z/χ²*	*P*
Age, *M (Q_1_, Q_3_)*	55 (46, 63)	53 (46, 62)	55 (46, 63)	–0.49	0.627
Gender, *n* (%)				6.42	0.011
Male sex	170 (39.81)	100 (45.66)	70 (33.65)		
Female sex	257 (60.19)	119 (54.34)	138 (66.35)		
Overweight/Obesity, *n* (%)	275 (64.40)	130 (59.36)	145 (69.71)	4.99	0.026
FS-14, *n* (%)				43.58	<0.001
Mild	192 (44.96)	127 (57.99)	65 (31.25)		
Moderate	156 (36.53)	74 (33.79)	82 (39.42)		
Severe	79 (18.50)	18 (8.22)	61 (29.33)		
Smoking/Alcohol history, *n* (%)	137 (32.08)	64 (29.22)	73 (35.10)	1.69	0.194
Coffee/Tea history, *n* (%)	247 (57.85)	119 (54.34)	128 (61.54)	2.27	0.132
TST, *M (Q_1_, Q_3_)*	367.00 (314.00, 443.25)	367.00 (313.00, 449.35)	367.00 (314.38, 433.50)	–0.67	0.501
SE, *M (Q_1_, Q_3_)*	0.80 (0.67, 0.93)	0.82 (0.68, 0.94)	0.78 (0.67, 0.91)	–1.94	0.052
Sleep latency, M *(Q_1_, Q_3_)*	23.10 (13.00, 55.00)	22.00 (12.00, 49.00)	24.20 (13.00, 60.25)	–0.98	0.328
REM latency, *M (Q_1_, Q_3_)*	80.00 (28.50, 217.00)	76.00 (30.50, 170.50)	82.00 (26.75, 239.12)	–0.87	0.386
Frequency of awakenings, *M (Q_1_, Q_3_)*	5.00 (2.00, 15.00)	5.00 (3.00, 13.00)	4.00 (1.00, 18.25)	–2.58	0.01
N3%, *M (Q_1_, Q_3_)*	30.39 (15.12, 51.19)	35.43 (16.63, 61.22)	25.30 (14.57, 42.75)	–3.48	<0.001
Daily light exposure, *M (Q_1_, Q_3_)*	60.00 (30.00, 90.00)	60.00 (40.00, 90.00)	50.00 (30.00, 82.50)	–2.62	0.009
Screen time before bedtime, *M (Q_1_, Q_3_)*	100.00 (60.00, 120.00)	80.00 (60.00, 120.00)	100.00 (60.00, 120.00)	–3.07	0.002
Total shift work duration, *M (Q_1_, Q_3_)*	2.00 (0.00, 5.00)	1.00 (0.00, 5.00)	2.00 (0.00, 5.00)	–2.38	0.017
Work schedules, *n* (%)				90.98	<0.001
Regular day shift	154 (36.07)	111 (50.68)	43 (20.67)		
Retirement	95 (22.25)	65 (29.68)	30 (14.42)		
8-hour rotating shifts	66 (15.46)	18 (8.22)	48 (23.08)		
12-hour rotating shifts	56 (13.11)	11 (5.02)	45 (21.63)		
24-hour shifts	56 (13.11)	14 (6.39)	42 (20.19)		
Shift work rotation frequency, *n* (%)				90.24	<0.001
None	249 (58.31)	176 (80.37)	73 (35.10)		
Slow	80 (18.74)	21 (9.59)	59 (28.37)		
Medium	60 (14.05)	14 (6.39)	46 (22.12)		
Fast	38 (8.90)	8 (3.65)	30 (14.42)		

This table compares baseline characteristics and candidate predictors between patients with CFS with and without comorbid CRSWDs in the training cohort. Continuous variables are presented as median *(Q_1_, Q_3_)*, and categorical variables as *n* (%).

### 3.2 Univariable and multivariable logistic regression analysis

Using the presence of CRSWDs as the dependent variable (CRSWDs: 1 = yes, 0 = no), we performed univariable logistic regression for each candidate factor. The univariable analysis indicated that several factors were significantly associated with the occurrence of CRSWDs in patients with CFS (*P* < 0.05). These included shift-work rotation frequency (OR = 2.649), FS-14 (OR = 2.471), female sex (OR = 1.657), overweight/obesity (OR = 1.576), pre-sleep screen time (OR = 1.007 per minute of use), SWS (N3%) (OR = 0.981 per 1% increase), and shift-work schedule type. Relative to regular day shift, 8-hour rotating shifts, 12-hour rotating shifts, and 24-hour shifts were associated with higher odds of CRSWDs (OR = 6.884, 10.560, and 7.744, respectively; all *P* < 0.001). In contrast, age, smoking/alcohol history, coffee/tea consumption history, daily light exposure duration, TST, sleep efficiency, sleep latency, REM latency, WASO, and total years of shift work were not significantly associated with CRSWDs in the univariable analysis (*P* > 0.05). Detailed univariable regression results are presented in **Table 2**.

**Table 2:** Univariable logistic regression analysis for factors associated with CRSWDs in CFS (model cohort, *n* = 427).

Variables	*β*	SE	*Z*	*P*	OR (95% CI)
Age	0.004	0.008	0.503	0.615	1.004 (0.988–1.021)
Gender					
Male sex	Ref				
Female sex	0.505	0.200	2.526	0.012	1.657 (1.120–2.451)
Overweight/obesity	0.455	0.204	2.227	0.026	1.576 (1.056–2.351)
FS-14	0.904	0.142	6.350	<0.001	2.471 (1.869–3.266)
Alcohol consumption and smoking status	0.270	0.208	1.298	0.194	1.310 (0.871–1.968)
Coffee and tea consumption	0.296	0.197	1.505	0.132	1.345 (0.914–1.977)
Daily light exposure	-0.005	0.003	–1.863	0.062	0.995 (0.990–1.000)
Screen time before bedtime	0.007	0.002	3.192	0.001	1.007 (1.003–1.011)
TST	0.000	0.001	0.159	0.873	1.000 (0.998–1.002)
SE	-0.750	0.530	–1.414	0.157	0.472 (0.167–1.336)
Sleep latency	0.002	0.003	0.825	0.409	1.002 (0.997–1.007)
REM sleep latency	0.001	0.001	1.278	0.201	1.001 (0.999–1.003)
WASO	0.009	0.008	1.063	0.288	1.009 (0.992–1.026)
N3%	-0.019	0.005	–4.115	<0.001	0.981 (0.973–0.990)
Total shift-work duration	0.000	0.013	0.006	0.995	1.000 (0.974–1.027)
Work schedules					
Regular day shift	Ref				
Retirement	0.175	0.285	0.615	0.538	1.191 (0.682–2.081)
8-hour rotating shifts	1.929	0.330	5.853	<0.001	6.884 (3.608–13.134)
12-hour rotating shifts	2.357	0.381	6.182	<0.001	10.560 (5.002–22.297)
24-hour shifts	2.047	0.357	5.733	<0.001	7.744 (3.846–15.593)
Shift-work rotation frequency	0.974	0.126	7.724	<0.001	2.649 (2.069–3.393)

This table presents the results of univariable logistic regression analysis for candidate factors associated with comorbid CRSWDs in patients with CFS. Variables identified in this analysis were considered for inclusion in the subsequent multivariable logistic regression model.

To avoid circular reasoning caused by shift work serving as both a predictor variable and a diagnostic criterion for CRSWDs, we made a fundamental correction to the outcome definition specifically for all regression analyses. For statistical modeling purposes only, the diagnosis of CRSWDs was based exclusively on two objective, shift-work-independent criteria: (1) documented circadian rhythm disturbances on polysomnography or 7-day sleep diaries (reduced rhythm amplitude, phase shift ≥ 2 hours, or frequent nocturnal awakenings); and (2) extreme chronotype on the MEQ-SA questionnaire (score ≤ 41 or ≥ 59). The criterion of “chronic maladaptive sleep schedule” (including shift work history) was completely excluded from the outcome definition to ensure that shift work was evaluated purely as an independent risk factor and did not contribute artificially to the classification of the outcome. This methodological adjustment was clearly distinguished from the standard clinical diagnostic criteria for CRSWDs used for patient enrollment, as detailed in Section 2.4.3.

Next, all variables that were significant in the univariable analysis were entered into a multivariable logistic regression model (using backward stepwise selection) to identify factors independently associated with CRSWDs. Multicollinearity among independent variables was assessed, and all VIF values were < 2.0, well below the conventional threshold of 5, indicating no severe multicollinearity.

**Figure 2:**
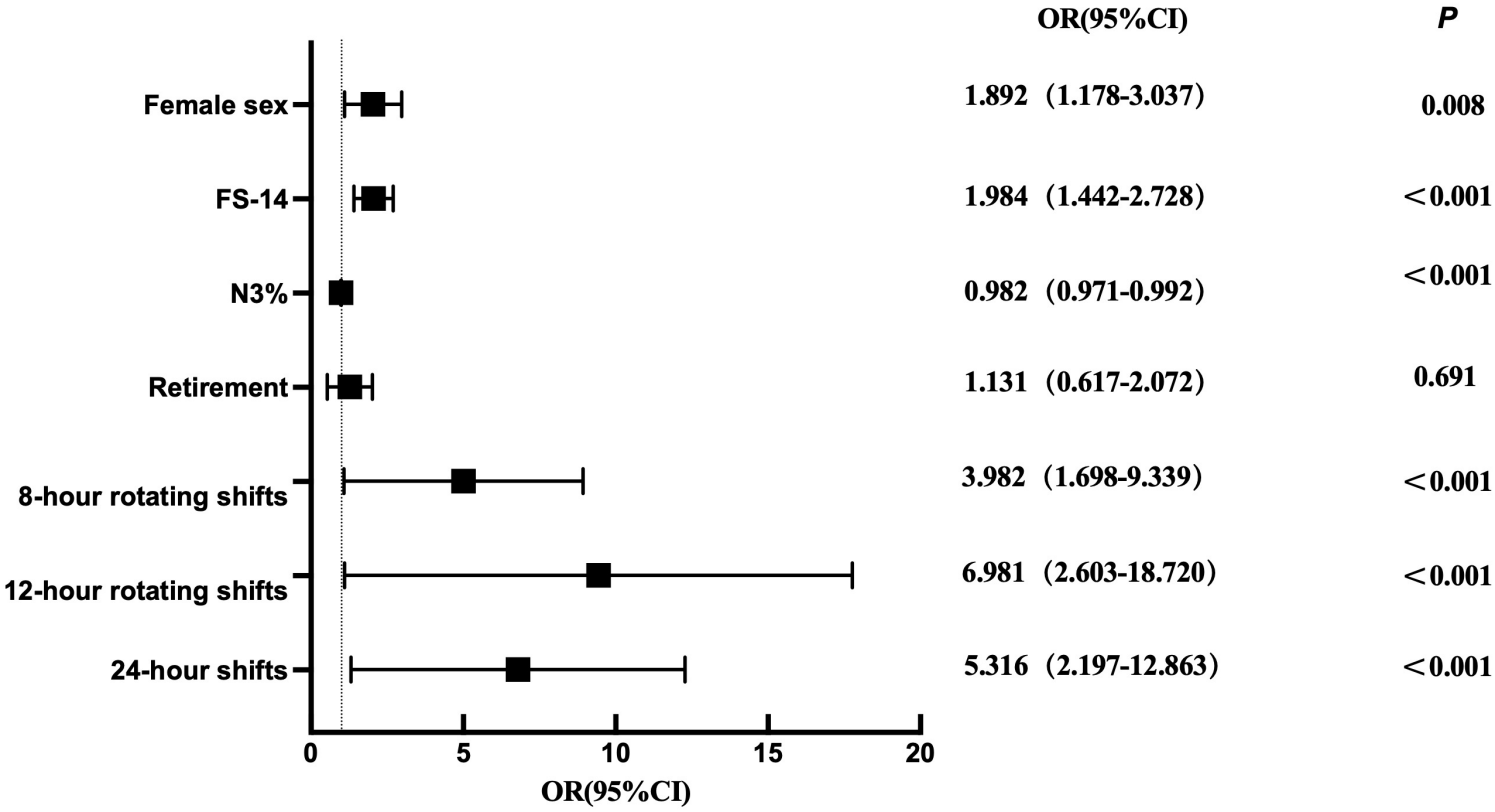
Forest plot of factors independently associated with comorbid CRSWDs in patients with CFS. This image presents the independent factors retained in the final multivariable logistic regression model for CRSWDs in patients with CFS.

The multivariable analysis showed that shift-work schedule type was the factor most strongly associated with CRSWDs in patients with CFS. Specifically, a 12-hour rotating shift schedule was associated with the highest odds of CRSWDs, followed by 24-hour shifts and 8-hour rotating shifts. In addition, higher fatigue severity and female sex were independently associated with higher odds of comorbid CRSWDs in patients with CFS. In contrast, an increase in SWS (N3%) was independently associated with lower odds of CRSWDs, with each percentage increase in N3% correlating with a reduced incidence of CRSWDs. These results are summarized in **Table 3**. **Figure 2** illustrates the multivariable results as a forest plot.

**Table 3:** Multivariable logistic regression analysis of factors associated with CRSWDs in CFS patients.

Variables	*B*	SE	Wald	*P*	OR	95% CI lower	95% CI upper
Gender							
Male sex	Ref						
Female sex	0.638	0.242	6.968	0.008	1.892	1.178	3.037
FS-14	0.685	0.163	17.750	<0.001	1.984	1.442	2.728
N3%	-0.018	0.005	11.340	<0.001	0.982	0.971	0.992
Work schedules**			22.583	<0.001			
Regular day shift	Ref						
Retirement	0.123	0.309	0.158	0.691	1.131	0.617	2.072
8-hour rotating shifts	1.382	0.435	10.099	<0.001	3.982	1.698	9.339
12-hour rotating shifts	1.943	0.503	14.907	<0.001	6.981	2.603	18.720
24-hour shifts	1.671	0.451	13.737	<0.001	5.316	2.197	12.863
Constant	-2.817	0.637	19.541	<0.001	0.060		

This table presents the results of the multivariable logistic regression analysis for factors independently associated with comorbid CRSWDs in patients with CFS.

**Shift schedule type was a categorical variable and was entered into the logistic regression model using indicator (dummy) variables, with “Regular day shift” as the reference category (0, Ref.). “Retirement” (1; Retired individuals were included as a separate category to account for potential differences in sleep-wake schedules compared to regular day shift workers.), “8-hour rotating shifts”(2), “12-hour rotating shifts” (3), and “24-hour shifts” (4) were each compared with the reference group, and odds ratios (OR) with 95% confidence intervals (CIs) were reported.

### 3.3 Predictive model development

#### 3.3.1 Nomogram risk prediction model

**Figure 3:**
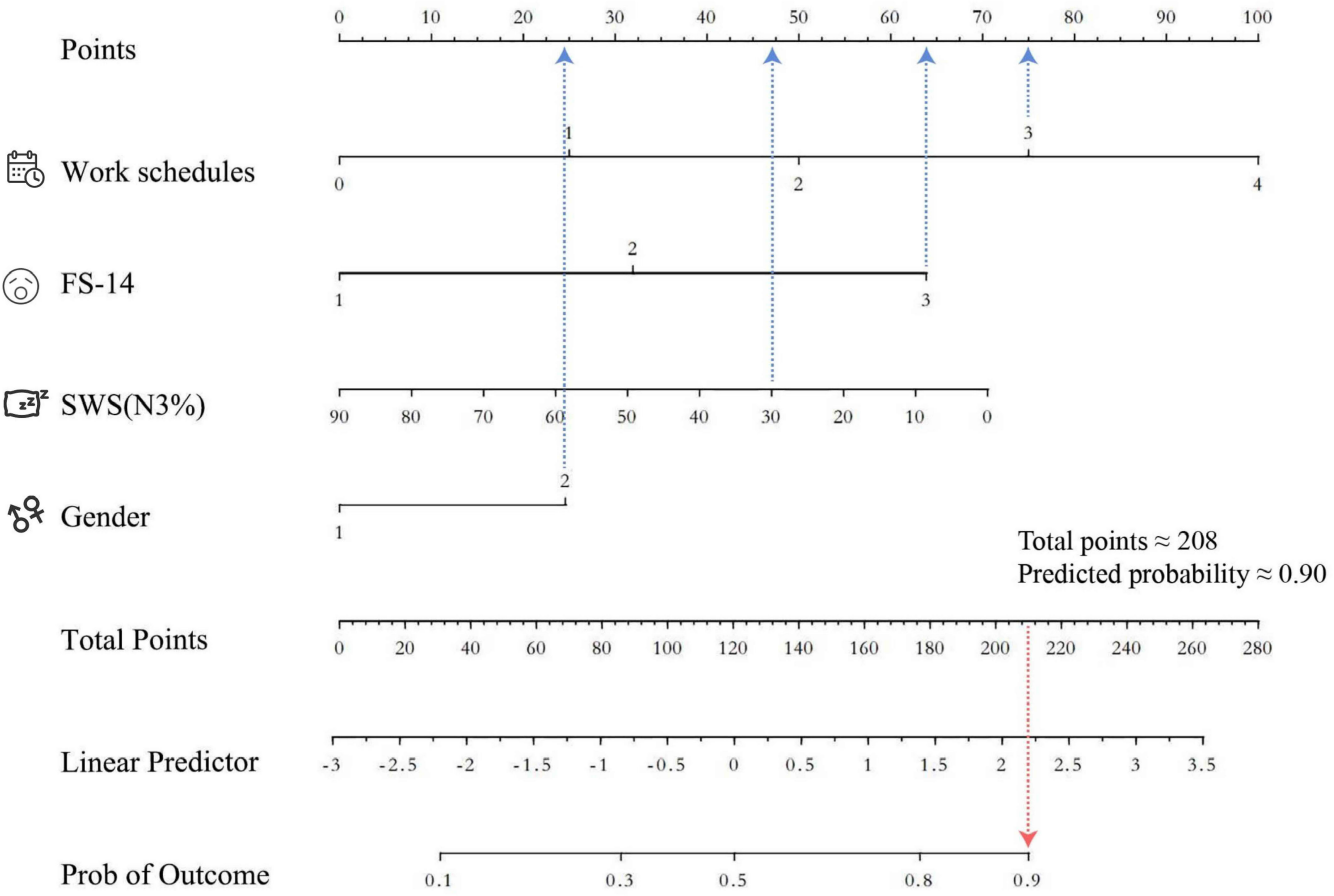
Nomogram for predicting comorbid CRSWDs in patients with CFS. This image presents a nomogram constructed from the final multivariable logistic regression model for predicting the risk of comorbid circadian rhythm sleep-wake disorders (CRSWDs) in patients with chronic fatigue syndrome (CFS). Simple illustrative icons are aligned on the left side of each predictor row to facilitate visual identification of variables and improve the interpretability of the nomogram. The nomogram includes work schedules, fatigue severity (Fatigue Scale-14, FS-14), slow-wave sleep (SWS, N3%), and gender. Each predictor is assigned a point value based on its proportional regression coefficient. The variable with the largest absolute coefficient (Work schedules) sets the scale maximum (100 points), and the point values for the remaining variables (e.g., N3%) are scaled relative to this maximum. The total points are then summed to estimate the individual probability of the outcome. Dashed index lines demonstrate how each predictor is mapped to the Points axis, summed as Total Points, and converted into the predicted probability of comorbid CRSWDs.

Based on the above multivariable logistic regression results, a nomogram model was constructed to predict the risk of CRSWDs comorbidity in individual CFS patients (**Figure 3**). The nomogram assigns a point value to each predictor (e.g., gender, fatigue severity, N3%, and type of shift schedule) according to its contribution to risk, and the total score corresponds to a predicted risk of having a CRSWD. The total nomogram score ranges from 0 to 280, with higher scores indicating a greater risk. For instance, a total score ≤ 29 points corresponds to an estimated risk of ≤ 10% for a CFS patient to have a comorbid CRSWD. In contrast, a total score of about 208 points predicts approximately a 90% probability of CRSWDs.

#### 3.3.2 Evaluation of the predictive nomogram

**Figure 4:**
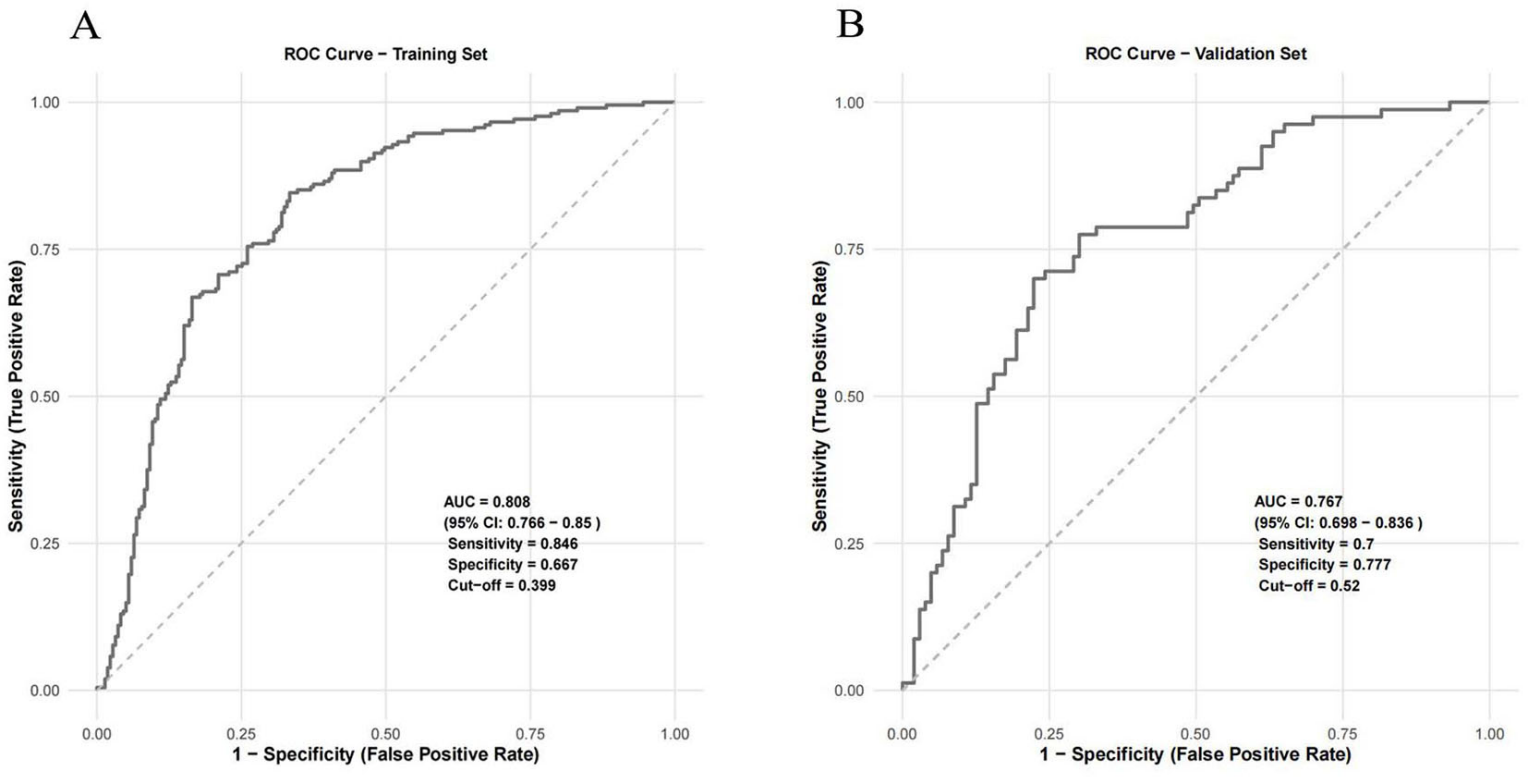
**(A, B)** ROC curves for the prediction of comorbid CRSWDs in patients with CFS. The ROC curves were used to assess model discrimination in the training and validation cohorts. The x-axis shows specificity and the y-axis shows sensitivity. The model yielded an AUC of 0.808 in the training cohort and 0.767 in the validation cohort. Panel **(A)** presents the ROC curve for the training cohort, with a sensitivity of 0.846, a specificity of 0.667, and an optimal cut-off value of 0.399. Panel **(B)** presents the ROC curve for the validation cohort, with a sensitivity of 0.700, a specificity of 0.777, and an optimal cut-off value of 0.520.

**Figure 5:**
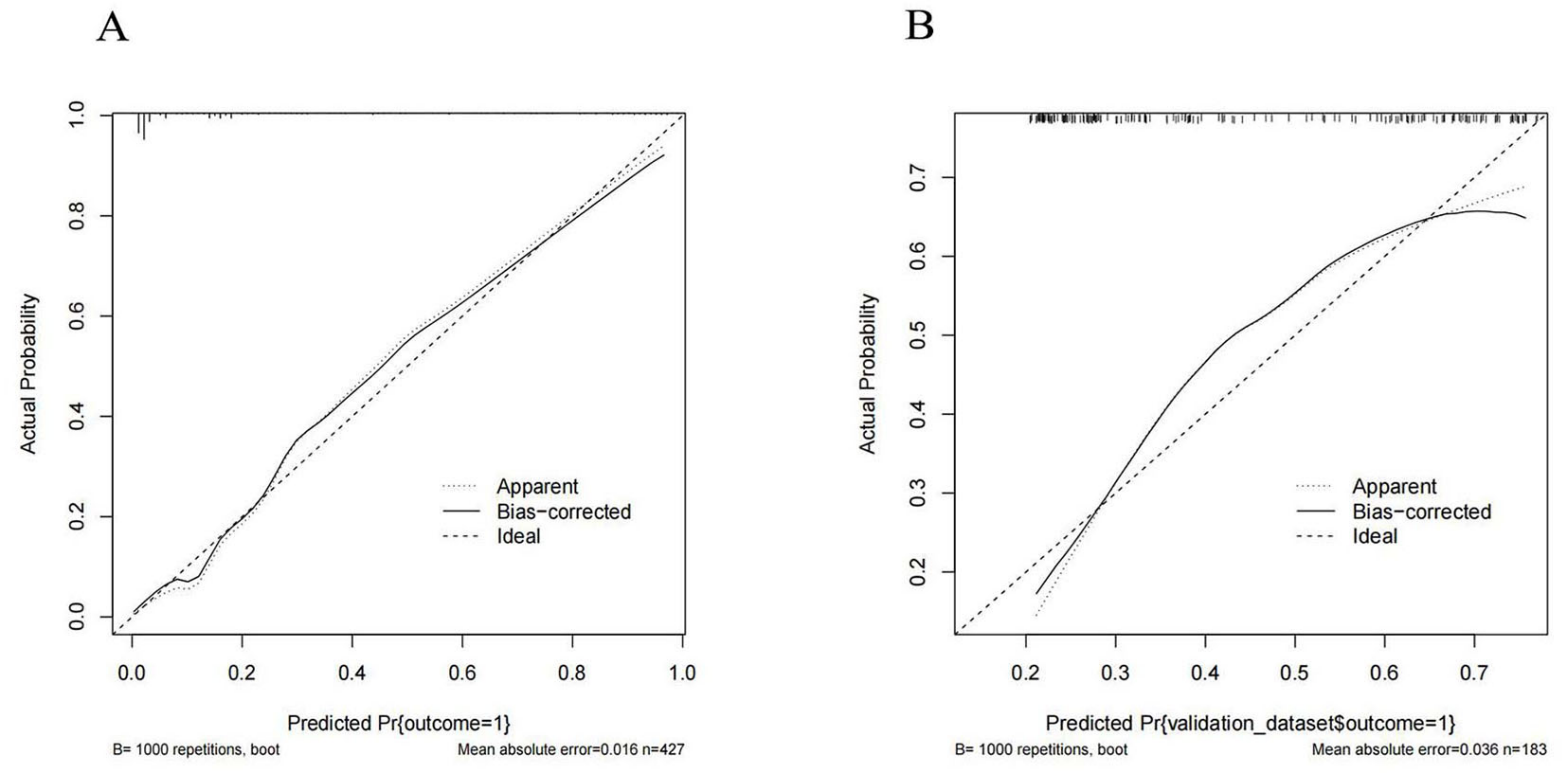
**(A, B)** Calibration curves for the prediction model of comorbid CRSWDs in patients with CFS. The curves depict the agreement between predicted and observed risk in the training and validation cohorts. The dashed diagonal line denotes ideal calibration, while the apparent and bias-corrected lines represent the original and bootstrap-corrected model performance, respectively. Panel **(A)** corresponds to the training cohort and Panel **(B)** to the validation cohort.

The model’s discrimination and calibration were assessed using the ROC curve and the Hosmer-Lemeshow test. ROC analysis (**Figures 4A, B**) showed that the AUCs in the training and validation cohorts were 0.808 (95% CI: 0.766-0.850) and 0.767 (95% CI: 0.698-0.836), respectively, both exceeding 0.7, indicating good discriminatory performance of the model for identifying CFS patients at risk of comorbid CRSWDs. The Hosmer-Lemeshow test results (**Figures 5A, B**) suggested good model fit.

**Figure 6:**
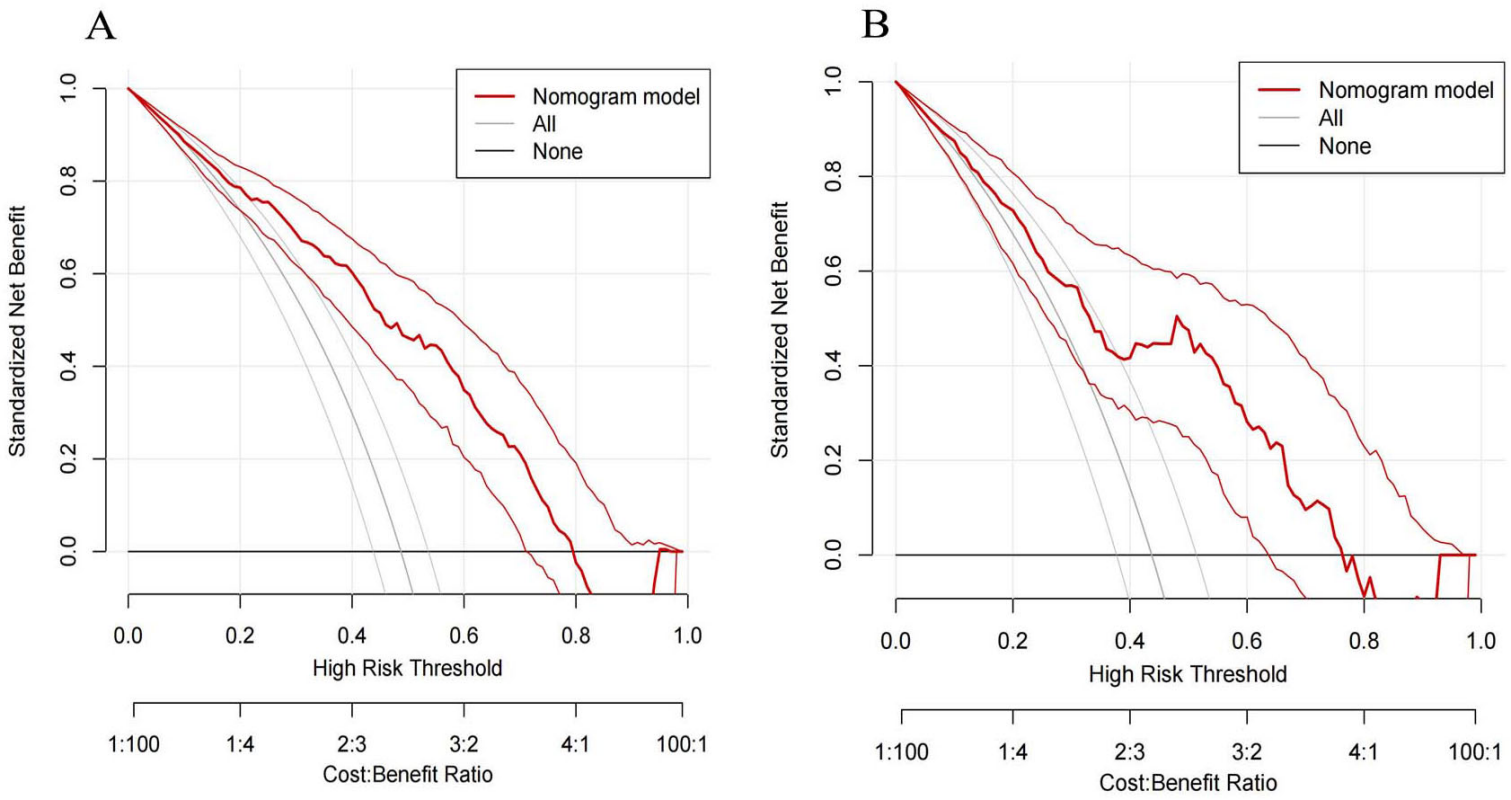
**(A, B)** Decision curve analyses for the prediction model of comorbid CRSWDs in patients with CFS. The plots present model net benefit across threshold probabilities in the training and validation cohorts. The red curve represents the nomogram, the grey line the treat-all strategy, and the black horizontal line the treat-none strategy. Panel **(A)** corresponds to the training cohort and Panel **(B)** to the validation cohort.

DCA was performed to evaluate the clinical utility of the model. The DCA curves indicated that, within the range of risk threshold probabilities in the training cohort (**Figure 6A**) and the validation cohort (**Figure 6B**), using this model to identify CFS patients with comorbid CRSWDs and implement interventions would yield a positive net benefit.

## 4 Discussion

This study developed a multivariable logistic regression model and a corresponding prediction tool to characterize factors associated with comorbid CRSWDs in patients with CFS. The findings suggest that CRSWDs in patients with CFS are associated with a complex, interacting pathological network involving work-schedule characteristics, fatigue severity, sleep architecture and demographic characteristics. These findings highlight the clinical relevance of circadian rhythm disruption on the quality of life and disease management of patients with CFS, and provide a foundation for further investigation of the underlying mechanisms.

Specifically, our findings highlight that irregular shift-work schedules and fatigue severity were the factors most strongly associated with comorbid CRSWDs in patients with CFS. Among the different shift work types, the 12-hour rotating shifts showed the strongest association with higher odds of CRSWDs (OR = 6.981), followed by the 24-hour shifts (OR = 5.316) and the 8-hour rotating shifts (OR = 3.982), indicating that unconventional rotating shift schedules were markedly associated with higher odds of CRSWDs. By contrast, neither the cumulative duration of shift work nor the frequency of shift rotation showed a statistically significant association with CRSWDs in this study. This may be because shift schedule type (e.g., 12-hour rotating shifts) has a more pronounced acute effect on circadian alignment than cumulative exposure, and the strong collinearity between shift schedule type and rotation frequency may have masked the independent effect of the latter. On the individual patient level, fatigue severity was also independently associated with higher odds of CRSWDs, second only to shift-work schedule type in the model (OR = 1.984 for each higher category of fatigue), suggesting a tight link between severe fatigue symptoms and circadian rhythm disturbances in CFS. Conversely, we found that a higher proportion of SWS (N3%) was independently associated with lower odds of CRSWDs (OR = 0.982). This emphasizes the importance of maintaining sufficient deep sleep for stabilizing the body’s circadian rhythms.

Notably, gender remained a significant predictor in the multivariable model, after adjustment for other covariates, female patients had higher odds of comorbid CRSWDs than male patients (OR = 1.892, 95% CI: 1.178-3.037; *P* = 0.008), whereas variables commonly considered relevant, such as age and TST, were not retained. This pattern suggests that, within this specific CFS population, vulnerability to circadian rhythm disruption may be more strongly related to fatigue burden and shift-work-related environmental stressors rather than age-related changes or overall sleep duration. Although insufficient daytime light exposure and pre-sleep electronic screen use were also not retained in the final model, their potential biological relevance should not be discounted. Both exposures can suppress physiological normal nocturnal melatonin secretion and diminish endogenous sleep drive. Their exclusion is likely attributable to collinearity with major predictors (e.g., shift-work schedule and fatigue severity), which may have attenuated their apparent independent effects after adjustment. Additionally, relative homogeneity of light-exposure patterns across the study population may have further reduced the ability to detect statistically significant effects.

Our findings align with prior evidence that night work and rotating schedules perturb endogenous circadian timing, supporting the view that misalignment between imposed work schedules and intrinsic circadian regulation is the principal external driver of rhythm disturbance. This highlights the need for clinicians and employers to pay close attention to work schedule arrangements. Adjusting and optimizing shift work systems may be an important public health strategy associated with lower prevalence of circadian rhythm disorders in high-risk populations (8, 25, 26). Similarly, the fact that fatigue severity emerged as a major risk factor in our study reinforces the intertwined relationship between fatigue and circadian dysregulation in CFS. It is plausible that severe fatigue in CFS may both result from circadian rhythm disturbance and further exacerbate it. For example, prolonged severe fatigue might disturb the hypothalamic-pituitary-adrenal (HPA) axis and alter energy metabolism, which in turn may be associated with destabilized endogenous circadian rhythms (27, 28). This suggests a potential bidirectional feedback loop where circadian disruption worsens fatigue, and heightened fatigue feeds back to further disrupt circadian homeostasis. These results lend support to this bidirectional mechanism, which has been hinted at in other studies examining fatigue and biological rhythm imbalances.

Recent research has provided additional evidence that abnormal sleep-wake rhythms are strongly associated with the pathogenesis of CFS, a phenomenon further underscored by large-scale epidemiological observations, such as the increased risk of CFS following viral infections like COVID-19 (29). A large nationwide survey of SARS-CoV-2 convalescents in China demonstrated that persistent symptoms such as fatigue were prevalent approximately five months post-infection (30). Concurrently, accumulating clinical evidence on long COVID has underscored the frequent co-occurrence of fatigue, cognitive impairment, and autonomic dysfunction following viral infections (31). Although long COVID and CFS are clinically distinct conditions, their overlapping manifestations suggest that post-infectious fatigue syndromes may share features of multisystem dysregulation. Neurological imaging and metabolomic studies have shown that CFS patients can exhibit HPA axis hypoactivity, delayed melatonin rhythm phase, and abnormal neural connectivity between the hippocampus and prefrontal cortex, among other changes. These physiological alterations suggest that circadian imbalance may be associated with accelerated worsening of fatigue and cognitive dysfunction in CFS through pathways involving energy metabolism disruption, neuroinflammatory responses, and impaired mood regulation (4, 32).

Patients with CFS frequently exhibit disturbances in thermoregulation and autonomic function, often manifested as increased sympathetic activity during the daytime and inadequate nocturnal withdrawal. These alterations may be associated with further destabilization of sleep homeostasis and disruption of endogenous circadian alignment (33, 34). Evidence also indicates that sustained insufficient daytime light suppresses melatonin production and delays its nocturnal peak, thereby potentially weakening sleep drive and contributing to persistent fatigue and cognitive decline (35–37). Although daytime light exposure and pre-sleep screen use were not independently associated with CRSWDs in our multivariable model, their well-documented biological effects on circadian rhythms suggest they remain important targets for intervention. Their exclusion from the final model is likely attributable to collinearity with major predictors (e.g., shift-work schedule type) and relative homogeneity of light-exposure patterns in our study population, as noted earlier. Accordingly, modifiable environmental zeitgebers, such as optimizing light-exposure patterns, adjustments to work schedules, and limiting evening screen use, may represent promising targets for future interventions aimed at mitigating CRSWDs in individuals with CFS.

Moreover, the risk prediction model derived from the multivariable analysis showed strong overall performance, with AUC values exceeding 0.7 in both the training and validation cohorts, indicating good discriminative ability. The Hosmer-Lemeshow test further supported satisfactory calibration, and decision curve analysis demonstrated a meaningful net clinical benefit across a range of clinically relevant threshold probabilities. Collectively, these results underscore the model’s potential utility as a quantitative tool for identifying patients with CFS who have a higher estimated probability of comorbid CRSWDs.

Beyond risk prediction alone, this model possesses practical utility for early screening and targeted sleep health management in clinical and occupational health settings. For instance, this nomogram could be integrated into routine occupational health screenings for workers on irregular schedules. Occupational health physicians could use the scoring system to proactively identify CFS patients at high risk for CRSWDs and preemptively implement targeted interventions, such as sleep hygiene counseling, timed light therapy, or optimized shift-rotation schedules. From a public health perspective, these findings support the incorporation of sleep-related risk assessments into health promotion strategies for vulnerable populations.

This study has several limitations. First, because of the retrospective design, causal relationships could not be established. It remains unclear whether circadian disruption preceded and aggravated fatigue, or whether more severe fatigue contributed to circadian disturbance; our findings represent associations rather than causations. Second, participants were recruited from a single clinical center, limiting the generalizability of the findings. Third, residual confounding may arise from unmeasured variables, including psychiatric symptom severity, physical activity levels, socioeconomic status, occupational category, and unrecorded occasional medication use. Although we strictly excluded all patients with routine use of strong sedatives, hypnotics, and melatonin agonists, occasional use of over-the-counter sedatives, first-generation antihistamines, or low-dose anxiolytics was not systematically recorded in the electronic medical records. Such medications have only mild and transient effects on sleep architecture and circadian function, and their impact on our core findings is expected to be minimal. However, they represent a potential source of residual confounding that could slightly attenuate the observed associations. Finally, the model was mainly based on questionnaire data and polysomnographic measures and did not include objective markers of endogenous circadian status, such as melatonin secretion profiles or core body temperature rhythms. Additionally, we excluded the “chronic maladaptive sleep schedule” criterion from the outcome definition in our regression analyses to avoid circular reasoning. While this approach eliminates bias in the association between shift work and CRSWDs, it may slightly underestimate the overall prevalence of CRSWDs in this population.

## Data Availability

The original contributions presented in the study are included in the article/supplementary material. Further inquiries can be directed to the corresponding author.
